# Acute urinary morbidity after a permanent ^125^I implantation for localized prostate cancer

**DOI:** 10.1093/jrr/rru065

**Published:** 2014-07-25

**Authors:** Saiji Ohga, Katsumasa Nakamura, Yoshiyuki Shioyama, Katsunori Tatsugami, Tomonari Sasaki, Takeshi Nonoshita, Tadamasa Yoshitake, Kaori Asai, Hideki Hirata, Seiji Naito, Hiroshi Honda

**Affiliations:** 1 Department of Clinical Radiology, Graduate School of Medical Sciences, Kyushu University, 3-1-1 Maidashi, Higashi-ku, Fukuoka, Japan; 2 Kyushu International Heavy Ion Beam Cancer Treatment Center, 415 Harakoga, Tosu, Saga, Japan; 3 Department of Urology, Graduate School of Medical Sciences, Kyushu University, 3-1-1 Maidashi, Higashi-ku, Fukuoka, Japan; 4 School of Health Sciences, Faculty of Medicine, Kyushu University, 3-1-1 Maidashi, Higashi-ku, Fukuoka, Japan

**Keywords:** acute urinary morbidity, ^125^I implantation, prostate cancer

## Abstract

We evaluated the predictive factors of acute urinary morbidity (AUM) after prostate brachytherapy. From November 2005 to January 2007, 62 patients with localized prostate cancer were treated using brachytherapy. The ^125^Iodine (^125^I) seed-delivering method was a modified peripheral pattern. The prescribed dose was 144 Gy. Urinary morbidity was scored at 3 months after implantation. The clinical and treatment parameters were analysed for correlation with AUM. In particular, in this study, Du90 (the minimal dose received by 90% of the urethra), Dup90 (the minimal dose received by 90% of the proximal half of the urethra on the bladder side) and Dud90 (the minimal dose received by 90% of the distal half of the urethra on the penile side) were analysed. We found that 43 patients (69.4%) experienced acute urinary symptoms at 3 months after implantation. Of them, 40 patients had Grade 1 AUM, one patient had Grade 2 pain, and two patients had Grade 2 urinary frequency. None of the patients had ≥Grade 3. Univariate and multivariate analysis revealed that Du90 and Dup90 were significantly correlated with AUM. In this study, Du90 and Dup90 were the most significant predictors of AUM after prostate brachytherapy.

## INTRODUCTION

In Japan, the number of patients identified with early-stage prostate cancer has increased along with the rise in prostate-specific antigen (PSA) examinations. In early-stage prostate cancer patients, it is important to control the localized lesion in the prostate gland. Several groups have reported that prostate brachytherapy (PB) resulted in excellent outcomes for T1–2 prostate cancer patients [1–5]. The treatment outcomes following PB for early-stage prostate cancer compare well with prostatectomy and external beam radiotherapy [6]. Because the adverse effects of PB are reasonably acceptable [7–9] and the duration of hospitalization is short, the number of patients in Japan who undergo PB using radioactive ^125^iodine (^125^I) seeds has increased rapidly in recent years [10].

However, many patients who receive PB for prostate cancer develop some degree of acute urinary morbidity (AUM), the symptoms of which include urinary symptoms such as incontinence, problems with frequency, retention, hematuria and dysuria; the symptoms usually have reach maximum severity before 3 months and resolve within one year [11, 12]. Especially for patients who have no symptoms before PB, AUM becomes bothersome. It is thus very important to clarify the treatment parameters that may affect the severity of AUM. Some authors have reported a correlation between AUM and the dose of urethra in prostate gland [11–13]. Salem *et al*. reported that a V150 of the urethra (the percent urethral volume receiving 150% of the prescribed dose) >40% was a significant predictor of AUM [11]. Desai *et al*. reported that the urethral dose was significantly correlated with the frequency of AUM [12]. Akimoto *et al*. reported a correlation between the severity of AUM and the urethral dose in high-dose-rate (HDR) brachytherapy for prostate cancer [13]. Although some studies have demonstrated that the urethral dose is correlated with the occurrence of AUM, there are few reports on the relationship between the doses to the segments of the urethra and AUM [14]. In this study, we evaluated the predictive factors of AUM, focusing on the urethral segmental doses.

## MATERIALS AND METHODS

From November 2005 to January 2007, 62 patients with localized prostate cancer were treated using PB alone at Kyushu University Hospital. We obtained informed consents prior to PB from all patients. The patient characteristics are shown in Table 1. Patients who received additional external beam radiotherapy after PB were excluded from this study. Of the 62 patients, 34 received hormonal therapy before PB.

**Table 1.** Patient characteristics

		No.	Range (Median)
Age (years)			55–79 (69)
Stage	T1c	48	
	T2a	8	
	T2b	6	
Gleason score	3 + 3	46	
	3 + 4	16	
Prostate-specific antigen	≤10	57	
	>10	5	
Pre-treatment IPSS^a^			1–27 (8)

^a^IPSS = International Prostate Symptom Score.

At 1 month before implantation, all patients underwent a transrectal ultrasound-based pre-planning examination as a pre-plan for the evaluation of the prostate volume and the determination of the number of seeds that would be enough to surround the prostate gland with isodose lines of the prescribed dose. The modified peripheral method was used for the prostate implants. The prescribed dose was 144 Gy. At 24 h and at 1 month after implantation, all patients underwent computed tomography (CT) scans in order to determine the seeds that were actually delivered. On the CT at 1 month after implantation, a single physician contoured the prostate, the urethra and the rectum, and the dose distribution was calculated as the post-plan dosimetry. The pre-treatment/treatment characteristics are shown in Table 2.

**Table 2.** Pre-treatment/treatment characteristics

Variable	No.	Range	Median
Prostate volume at pre-plan (cm^3^)		14.3–35.8	26.1
Prostate volume at post-plan (cm^3^)		16.0–43.7	27.8
Number of needles		15–40	25
Number of seeds		59–98	75
Total seed activity (mCi)		19.47–32.34	24.75
Hormonal therapy	34 (54.8%)		

**Fig. 1.**
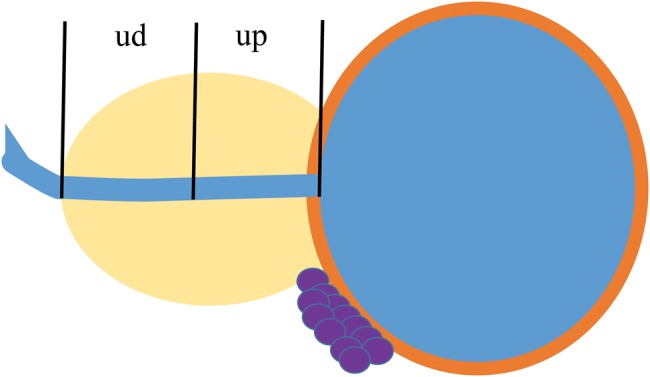
Prostatic urethra equally divided into two sections. ud = half urethra on penile side, up = half urethra on urinary bladder side.

In particular, the urethra was delineated by referring to the location of the urethral tube on the CT image acquired at 24 h after implantation. The Dp90 (the value of the minimal dose received by 90% of the prostate), Vp100 and Vp150 (the percent prostate volume receiving 100% and 150% of the prescribed dose, respectively) were calculated routinely. In addition, the Du5, Du90 (the value of the minimal dose received by 5% and 90% of the urethra) and the U200 (the percent urethral volume receiving 200% of the prescribed dose) were also calculated. For our analysis of the relationship between the doses to the segments of the urethra and AUM, the urethra was divided into the proximal and distal segments (Fig. 1). We also calculated the Dup90 and Dup5 (the values of the minimal dose received by 90% and 5% of the proximal urethra, respectively) and the Dud90 and Dud5 (the values of the minimal dose received by 90% and 5% of the distal urethra, respectively). Other clinical and treatment factors examined included the number of needles, the number of seeds, the International Prostate Symptom Score (IPSS), the prostate volume and hormonal therapy.

All patients were assessed at 3 months after PB, because AUM reaches a maximum before 3 months and after that tends to regress [12]. The AUM symptoms of hematuria, dysuria, incontinence, urinary frequency and urinary retention were examined. AUM was scored using the CTCAE ver. 3.0 (National Cancer Institute Common Terminology Criteria for Adverse Events Version 3.0). The IPSS (International Prostate Symptom Score) was assessed at the same time.

Differences between the median values of Dud and Dup were tested using the Mann–Whitney's U test in Du5 and Du90. Correlations between AUM and all factors were analysed using the logistic regression model on univariate and multivariate analyses. All analyses were two-sided, and *P-*values < 0.05 were considered significant.

## RESULTS

The median values of the prostate volumes at pre- and post-plan were 26.1 cm^3^ and 27.8 cm^3^, respectively. The prostate volumes at 1 month after PB were greater than those before PB (Table 2). The dosimetric parameters for all patients are shown in Table 3. The median values of Vp100, Vp150 and Dp90 of the prostate were 92.9%, 56.7% and 152.8 Gy, respectively. The median values of Du5, Du90 and U200 of the urethra were 204.5 Gy, 127.1 Gy and 0%, respectively. The median values of Dud5, Dud90, Dup5 and Dup90 were 208.1, 148.9, 198.7 and 135.0 Gy, respectively. The median values of Dud5 and Dud90 were greater than those of Dup5 and Dup90, respectively, and the differences were statistically significant.

**Table 3.** Dosimetric parameters at post-plan

Variable	Range	Mean	Median	*P*-value
Vp100 (%)	70.8–99.2	92.3	92.9	
Vp150 (%)	36.1–78.5	56.8	56.7	
Dp90 (Gy)	60–130.7	154	152.8	
Du5 (Gy)	103–200	206.8	204.5	
Dud5 (Gy)	103–200	213.7	208.1	] 0.02
Dup5 (Gy)	101–200	198	198.7	
Du90 (Gy)	46.4–127.8	124.7	127.1	
Dud90 (Gy)	55.5–177.2	151.9	148.9	] <0.001
Dup90 (Gy)	27–124.7	116	135	
U200 (%)	0–18	0.6	0	

Of the 62 patients, 43 (69.4%) experienced some degree of AUM. Table 4 shows the details of AUM. Grade 1 and 2 urinary morbidity were observed in 40 and three patients, respectively. One patient complained of Grade 2 pain, and two patients complained of Grade 2 urinary frequency. No patients experienced Grade 3 or more AUM.

**Table 4.** Acute urinary morbidity at 3 months after implantation

	Hematuria	Pain	Incontinence	Frequency	Dysuria
Grade 1	2	11	7	38	9
Grade 2	0	1	0	2	0

The univariate and multivariate analyses revealed that the Du90 and Dup90 were the significant predictors of AUM (Table 5). Apart from Du90 and Dup90, the clinical and treatment factors (i.e. the number of seeds or needles, prostate volume at pre-plan and post-plan, IPSS and hormonal therapy) were not significant predictors of AUM.

**Table 5.** Acute urinary morbidity at 3 months after PB (logistic regression analysis)

	UVA			MVA		
Variables	*P*	OR	95% CI	*P*	OR	95% CI
Age (years)	0.61	0.98	0.90–1.07	0.9	0.99	0.89–1.11
Number of seeds	0.56	0.98	0.93–1.04	0.23	1.12	0.94–1.37
Number of needles	0.58	0.97	0.86–1.09	0.55	0.93	0.74–1.15
Prostate volume at pre-plan (cm^3^)	0.32	0.96	0.87–1.04	0.76	1.06	0.71–1.63
Prostate volume at post-plan (cm^3^)	0.17	0.94	0.86–1.02	0.25	0.8	0.53–1.15
IPSS	0.34	0.96	0.86–1.04	0.86	0.99	0.88–1.10
Hormonal therapy	0.42	0.61	0.20–1.85			
Vp100 (%)	0.12	0.9	0.77–1.03			
Dp90 (Gy)	0.16	0.96	0.91–1.01			
Vp150 (%)	0.33	0.97	0.91–1.03			
Du90 (Gy)	**0.004**	0.95	0.91–0.99			
Dud90 (Gy)	0.46	1.01	0.99–1.03	0.77	1	0.97–1.03
Dup90 (Gy)	**0.001**	0.01	0.00027–0.19	**0.008**	0.95	0.92–0.98
Du5 (Gy)	0.35	1.01	0.99–1.04	0.79	1	0.97–1.04
Dud5 (Gy)	0.8	0.99	0.97–1.02			
Dup5 (Gy)	0.24	0.98	0.95–1.01			
U200 (%)	0.64	0.94	0.61–1.17			

UVA = univariate analysis, MVA = multivariate analysis, OR = odds ratio, 95% CI = 95% confidence interval.

## DISCUSSION

### Acute urinary morbidity

Permanent brachytherapy for localized prostate cancer provides excellent long-term control by delivering a high dose of radiation to the prostate gland. Several reports have described compatible outcomes for localized prostate cancer with radical prostatectomy, 3D conformal radiation therapy, or PB [7, 15–17]. Most patients who receive brachytherapy for localized prostate cancer develop some degree of AUM for about 1 year; this is one of the brachytherapy-related problems. Salem *et al*. reported that the rates of Radiation Therapy Oncology Group Grade 1 and 2 acute urinary toxicity after PB are 56% and 10%, respectively [11]. Tanimoto *et al*. reported that the incidence of urinary toxicity Grade 1 or higher at 1 and 6 months is 67% and 40%, respectively. Grade 2 or higher urinary toxicity is less than 1% at each timepoint [18]. In the present study, 43 (69.4%) of the 62 patients developed Grade 1 or 2 AUM, but there were no patients with Grade 3 or more AUM. These results were the same as those of other authors [11, 18]. Although AUM resolves within 12 months after implantation in most patients [19], some authors suggest that it is important to control AUM and to analyze the factors that predispose patients to AUM, because severe AUM might be related to late urinary toxicity [20, 21].

### Urethral dose

Crook *et al*. examined the predictive factors of acute urinary retention after permanent seed PB [22], and they found that the implant quality as determined by the D90, V100 and V200 of the prostate and the urethral dose does not predict acute urinary retention. In contrast, Desai *et al*. reported that the urethral dose appears to affect the frequency of AUM most significantly [12]. Urinary frequency is the most common symptom of acute urinary symptoms following PB [19]. In the present study, most of the patients with AUM also developed urinary frequency, and the urethral dose — especially the D90 of the urethra — was a significant predictive parameter for AUM, as was also found by Desai *et al*. [12].

In contrast, Steggerda *et al*. reported that the dose to a 1-cm^3^ hotspot in the bladder wall as well as the prostate volume is independently correlated with urinary morbidity symptom scores at 3 months and 6 months after implantation [23]. William *et al*. concluded that the peak in the IPSS response to PB is inversely correlated with the pre-implant IPSS and positively correlated with the number of seeds implanted above the prostatic base [20]. These findings support the results of our study, demonstrating that the most predictive factor was the half-urethral D90 on the bladder side (i.e. the Dup90). Therefore, AUM may be correlated with the doses to the urethra (and, in particular, to the doses to the urethra on the bladder side). In the present study, although the median value of Dud90 was more than that of Dup90, the former was not correlated with AUM in statistical analysis. The results of Thomas *et al*. are similar to those of the present study. Although they found that D10 and D90 at the apex are greater than those at the base, the prostatic base dose (but not the dose at the apex) is predictive of higher maximum IPSS during the first year after the implant. They suggested that variable radiation sensitivity of the segmented urethra is one possible hypothesis to explain their results [14]. Radiation sensitivities of the urethra on the bladder side may differ from those of other segmented urethra.

### Other factors

It has been suggested that the pre-treatment prostate volume is one of the predictors for AUM [11, 21]. However, Merrick *et al*. reported that the prostate size does not predict short-term urinary morbidity. The median prostate volume at pre-plan in their series was 34.3 cm^3^ [24]. Steggerda *et al*. analyzed lower urinary tract symptoms (LUTS) at 3 and 6 months after PB. They showed that of 43 patients with pre-treatment prostate volume < 44 cm^3^, only 11 suffered from severe LUTS, although of 31 patients with pre-treatment prostate volume > 44 cm^3^, 23 suffered from severe LUTS [23]. In the present study, because the prostate volumes of all patients at pre-plan were <40 cm^3^, we suspect that the pre-treatment prostate volume might not be correlated with AUM.

Keyes *et al*. concluded that an intraoperative needling technique (in addition to the effect of radiation to the prostate gland by implanted seeds) caused AUM [25]. Some authors reported that IPSS is a predictive factor for AUM [24–26]. Keyes *et al*. showed that a greater baseline IPSS was a significant predictor of acute urinary retention in a multivariate analysis [25]. Using the IPSS questionnaire, an overall risk of prolonged urinary retention following PB was reported by Terk *et al*.; they noted that patients with an IPSS of ≥20 had a 29% risk of retention, those with an IPSS of 10–19 had an 11% risk, and those with an IPSS <10 had a 2% risk [26]. In our study, although the number of needles, number of seeds, and IPSS were not correlated with AUM, but further investigations are necessary to test these findings.

## CONCLUSION

The results of the present study indicated that the Du90 and Dup90 were the most significant predictors of AUM after PB. We suggest that it is important to control not only the Du90 but also Dup90 values by precisely adjusting the seed location around the half of the urethra at bladder side to prevent severe AUM.

## FUNDING

Funding to pay the Open Access publication charges for this article was provided by a Health Labor Sciences Research Grant (H23-Sanjigan-Ippan-007).
